# Distribution, Pest Status and Fungal Associates of *Euwallacea* nr. *fornicatus* in Florida Avocado Groves

**DOI:** 10.3390/insects7040055

**Published:** 2016-10-14

**Authors:** Daniel Carrillo, Luisa F. Cruz, Paul E. Kendra, Teresa I. Narvaez, Wayne S. Montgomery, Armando Monterroso, Charlotte De Grave, Miriam F. Cooperband

**Affiliations:** 1 IFAS-Tropical Research and Education Center, University of Florida, Homestead, FL 33031, USA; luisafcruz@ufl.edu (L.F.C.); tnarvaez1@ufl.edu (T.I.N.); charlottedegrave@ufl.edu (C.D.G.); 2 Subtropical Horticulture Research Station, Agricultural Research Service, United States Department of Agriculture, Miami, FL 33158, USA; Paul.Kendra@ars.usda.gov (P.E.K.); wayne.montgomery@ars.usda.gov (W.S.M.); 3 Brooks Tropicals, LLC, Homestead, FL 33090, USA; Armando@brookstropicals.com; 4 Gembloux Agro-Bio Tech, University of Liège, Gembloux B-5030, Belgium; 5 Otis Laboratory, Plant Protection and Quarantine’s Science and Technology, Animal and Plant Health Inspection Service, United States Department of Agriculture, Buzzards Bay, MA 02542, USA; miriam.f.cooperband@aphis.usda.gov

**Keywords:** ambrosia beetles, symbiosis, *Fusarium*

## Abstract

Members of a complex of cryptic species, that correspond morphologically to the ambrosia beetle *Euwallacea fornicatus* (Eichhoff) (Coleoptera: Curculionidae: Scolytinae), were recently found attacking avocado (*Persea americana* Mill.) in Israel and California. In early 2016, an outbreak of another member of this species complex was detected infesting approximately 1500 avocado trees in an avocado orchard at Homestead, Florida. An area-wide survey was conducted in commercial avocado groves of Miami-Dade County, Florida to determine the distribution and abundance of *E.* nr. *fornicatus*, to identify different populations of *E.* nr. *fornicatus* and their fungal associates, and to assess the extent of damage to avocado trees. *Ewallacea* nr. *fornicatus* were captured in 31 of the 33 sampled sites. A sample of 35 beetles from six different locations was identified as *E*. nr. *fornicatus* sp. #2, which is genetically distinct from the species causing damage in California and Israel. Eleven fungal associates were identified: an unknown *Fusarium* sp., AF-8, AF-6, *Graphium euwallaceae, Acremonium* sp. *Acremonium morum*, *Acremonium masseei*, *Elaphocordyceps* sp. and three yeast species. The unknown *Fusarium* isolates were the most abundant and frequently found fungus species associated with adult beetles and lesions surrounding the beetle galleries. In addition to fungal associates, three bacteria species were found associated with adult *E.* nr. *fornicatus*. Visual inspections detected significant damage in only two orchards. A large number of beetles were captured in locations with no apparent damage on the avocado trees suggesting that *E.* nr. *fornicatus* are associated with other host(s) outside the groves or with dead trees or branches inside the groves. More research is needed to determine the potential threat *E.* nr. *fornicatus* and its fungal associates pose to the avocado industry and agricultural and natural ecosystems in Florida.

## 1. Introduction

Ambrosia beetles (Coleoptera: Curculionidae: Scolytinae) have recently emerged as important pests of avocado (*Persea americana* Mill.; Laurales: Lauraceae), because they have been found to vector plant pathogens to this food crop. Florida’s US $100 million avocado industry is being threatened by laurel wilt (LW), a lethal disease of members of the Lauraceae that is caused by *Raffaelea lauricola* T.C. Harr. Fraedrich & Aghayeva (Ophiostomatales: Ophiostomataceae). This disease is vectored by *Xyleborus glabratus* Eichhoff (Coleoptera: Curculionidae) and likely *X. volvulus* (F.) and *X. ferrugineus* (F.), which are more prevalent in the avocado production region of south Florida [1].

In addition, members of a complex of cryptic species that correspond morphologically to the ambrosia beetle, *Euwallacea fornicatus* (Eichhoff) (Coleoptera: Curculionidae: Scolytinae), the tea shot hole borer, have been found attacking avocado (*P. americana*) in Israel, California, and more recently, in Florida. A phylogenetic analysis of beetles and fungi collected from different parts of the world revealed the existence of a complex of cryptic species of *E. fornicatus* and Ambrosia Fusarium (AF), which is composed of at least five beetle species and nine fungal species that are all genetically distinct [2]. Two species, the polyphagous shot hole borer (PSHB) or *E.* nr. *fornicatus* sp. #1 per O’Donnell et al. 2015 [2], and *E.* nr. *fornicatus* sp. #5, have been reported to attack more than 300 host species in 58 plant families; including avocado in California [3]. *Euwallacea* nr. *fornicatus* sp. #1 also has been found attacking avocado in Israel [4]. *Euwallacea* nr. *fornicatus* sp. #1 forms symbiotic associations with *Fusarium euwallaceae*, *Graphium euwallaceae*, *Paracremonium pembeum*, and *E.* nr. *fornicatus* sp. #5 does so with *Fusarium* sp. AF-12 and *Graphium* sp. [2,3,5,6].

Populations of *E.* nr. *fornicatus* were first detected in a commercial avocado orchard in south Florida in 2012 [7]. Specimens collected from the location of the original detection were determined to be genetically distinct from California’s PSHB and *E*. nr. *fornicatus* sp. #5 [2,3] and subsequently referred to as *E*. nr. *fornicatus* sp. #2 [2]. Kasson et al. [8] identified two ambrosia fusaria (AF-6 and AF-8) cultivated by *E.* nr. *fornicatus* sp. #2. In addition, Cooperband et al. [9] observed that a single foundress produced her first adult female offspring within 22 days of gallery initiation at 24 °C, and averaged 24.7 adult female offspring within 6–7 weeks, and that the average sex ratio (% male) was 7.2%.

Subsequent surveys in south Florida from 2013 to 2015 revealed the presence of *E*. nr. *fornicatus* beetles in one mango and seven additional avocado orchards [10,11]. In all of these cases the *E*. nr. *fornicatus* populations were considered sparse and did not cause conspicuous damage to avocado or other crops. In early 2016, an outbreak of *E*. nr. *fornicatus* was detected in a 20-acre (8.1 ha) avocado orchard at Homestead, Florida, infesting approximately 1500 avocado trees in the grove [12]. The observed damage resembled that caused to avocado by PSHB in California. The beetles initially attacked junctions of small and mid-size shaded branches in the upper canopy, inoculating their fungal associates and causing localized lesions characterized by discoloration of the xylem tissue. The initial attack induced a conspicuous response by the plant, which produced white exudates referred to as “sugar volcanoes” [3]. The infestation progressed causing branch dieback, and copious breeding of the beetles occurred in decaying branches. Thereafter, attacks to larger limbs and the main trunk were observed. The similarity between the damage caused to avocado in Florida and California and the limited number of specimens used for initial characterization of the Florida species raised the question of whether other species of the *E*. nr. *fornicatus* complex were present in Florida. To address this question, an area-wide survey was conducted in the avocado production region of Miami-Dade County, Florida, which is located at the tip of the peninsula.

The objectives of this study were to determine the distribution and abundance of *E.* nr. *fornicatus* in south Florida’s commercial avocado groves, to identify different populations of *E*. nr. *fornicatus* and their fungal associates, and to assess the extent of damage caused to avocado trees.

## 2. Materials and Methods

### 2.1. Area-Wide Survey for E. nr. fornicatus Beetles in the Avocado Production Region of Miami-Dade County, Florida

**Figure 1:**
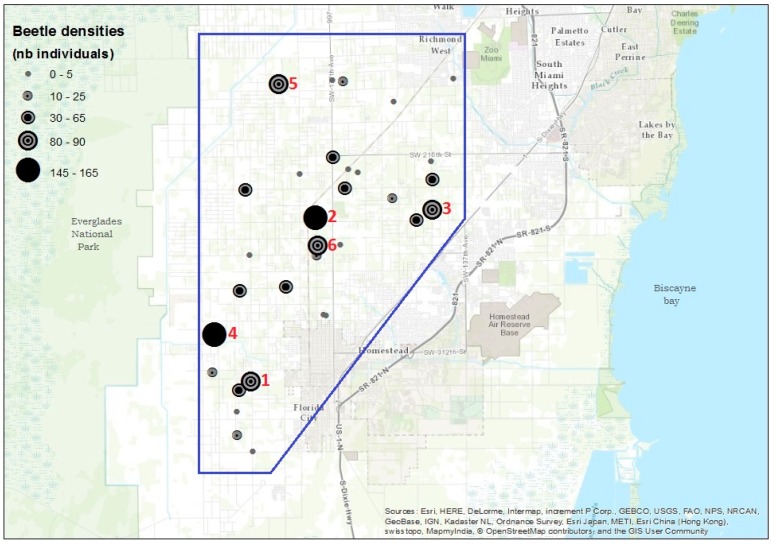
Distribution and abundance of *Euwallacea* nr. *fornicatus* in the avocado growing region in south Florida (enclosed in the blue polygon). Circles of different sizes represent the total number of *E.* nr. *fornicatus* beetles captured per avocado orchard.

The Florida avocado industry consists of approximately 7000 acres [13] located in the southernmost part of Miami-Dade County to the east of Everglades National Park and west of Biscayne Bay (Figure 1). Over 98 percent of the avocados grown commercially in Florida are grown in this area. Orchard size ranges from 0.1 to over 500 acres (0.04–202 ha), with 86 percent of the farms less than 15 acres (6 ha) [14]. Thirty-three avocado orchards distributed throughout the avocado growing region were selected as sites for the survey (Figure 1). The orchard in which an outbreak of *E*. nr. *fornicatus* was detected in January 2016 was included in the survey.

Two traps were deployed at each site. Traps consisted of two white sticky panels (23 × 28 cm, Sentry wing trap bottoms; Great Lakes IPM, Vestaburg, MI, USA) baited with lures containing quercivorol (ChemTica USA, Durant, OK, USA) and 50% α-copaene (Synergy Semiochemical Corp., Burnaby, BC, Canada)—the two best attractants currently known for *E.* nr. *fornicatus* [10,15]. Using wire hooks, traps were hung from avocado trees at approximately 1.5 m of height above the ground. The two traps in each site were separated by at least 25 m. The survey was initiated on 15 March 2016; two weeks later all traps were collected and transported to the Tropical Fruit Entomology Laboratory in Homestead, Florida for morphological identification of *E*. nr. *fornicatus*.

The abundance of *E*. nr. *fornicatus* in the orchard with a known outbreak was used as a reference to select five additional orchards with large beetle trapping numbers for molecular characterization of *E.* nr. *fornicatus* populations and their associated fungi. The six selected orchards were located in the center (*N* = 2) and along the edges (*N* = 4) of the avocado growing area at Homestead, Florida (Figure 1). At each of these orchards, visual inspections were conducted to assess the damage caused by *E.* nr. *fornicatus* beetles, and infested branches were collected from five trees. Five additional branches were collected from the orchard with the known outbreak. From each of the branches collected at each site, one female beetle and a sample of xylem tissue associated with the beetle gallery were removed for identification of the beetle species and fungal associates.

Beetles collected from excavated galleries were surface-disinfected with 95% ethanol for 1 min and subsequently washed with distilled sterile water three times. Heads were removed and heads and bodies were individually macerated in 100 µL of water. Total genomic DNA was extracted from beetle bodies using a modified cetyl trimethyl-ammonium bromide (CTAB) protocol [16]. Portions of the cytochrome oxidase subunit, elongation factor-1α, CAD protein, arginine kinase and mitochondrial 16S rDNA were amplified using oligonucleotide primers LCO1490 and HCO2198, ets149 and efa754, apCADforB2 and apCADrevlmod, forB2 and rev B2, LR-J-12961 and LR-N-13398 [17,18]. Nucleotide BLAST (Basic Local Alignment Search Tool) alignments were carried out for beetle identification.

### 2.2. Fungal Isolations from Plant Tissue and Beetles

Sections of discolored sapwood obtained from five branches per location were excised from the lesions surrounding beetle galleries and surface disinfected with ethanol and bleach. Pieces obtained from individual logs were independently plated onto potato dextrose agar supplemented with 0.1 g/L streptomycin (PDA^+^), and incubated at room temperature until the fungal growth was evident. For isolation of the beetle fungal communities, 40 µL of the beetle-head macerates and 100 µL of a 1:100 dilution were plated on PDA^+^ and malt extract agar amended with 0.6 g /L cycloheximide and 0.3 g/L streptomycin (CSMA). Colonies of representative morphotypes were counted and transferred onto new plates to obtain pure cultures.

DNA was extracted from the mycelium of pure cultures obtained for single-spore colonies for both plant and beetle isolates following a modified CTAB protocol [16]. The fungal isolates displaying *Fusarium*-like morphology were typed by sequencing portions of EF-1α amplified using primers EF1 and EF2 [19] and DNA-directed RNA polymerase II largest subunit RBP1 using primers F5/R8 and F8/R9 [2], and second largest subunit RBP2, 5f2/7cr and 7cf/11ar [20]. A portion of the nuclear large subunit (LSU) 28S ribosomal DNA was amplified for the identification of other fungal isolates using primers LR0R/LR5 [21]. *Graphium* spp. isolates identified by the above LSU primers were additionally typed using portions of the small subunit rDNA with primers NS1/NS4 [22]. Bacterial isolates were identified by amplifying and sequencing 16S rRNA using the primer set 63F/1387R [23]. PCR products were purified using ExoSAP-IT following the manufacturer’s protocols (Affimetrix) and sequenced in both directions. PCR conditions were as reported in [2,20] for *Fusarium* isolates, in [21,22] for other fungal isolates and in [23] for bacteria isolates. Sequencing was performed by Eurofins MWG Operon (Louisville, KY, USA). The NCBI BLAST was used to identify the nucleotide sequences.

## 3. Results

### 3.1. Area-Wide Survey for E. nr. fornicatus Beetles in the Avocado Production Region of Miami-Dade County, Florida

During the two-week survey, a total of 1164 *E*. nr. *fornicatus* individuals were captured in 31 of the 33 sampled sites (Figure 1). Trap captures ranged from 0 to 161 female beetles with an average of 35 ± 3.49 (mean ± standard error) beetles per avocado orchard. Damage and branch dieback were detected in only two of the 33 orchards including the orchard where an outbreak of *E*. nr. *fornicatus* had been detected previously.

All *E.* nr. *fornicatus* beetles collected from the six orchards with largest numbers of captures were identified as *E*. nr. *fornicatus* sp. #2 based on 99% identity of their five amplified gene segments COI, CAD, ArgK, 16SrRNA and EF-1α with the GeneBank accession numbers KM406731.1, KM406713.1, KM406700.1, KM406749.1, and KM406749.1.

### 3.2. Fungal Isolations from Plant Tissue and Beetles

**Figure 2:**
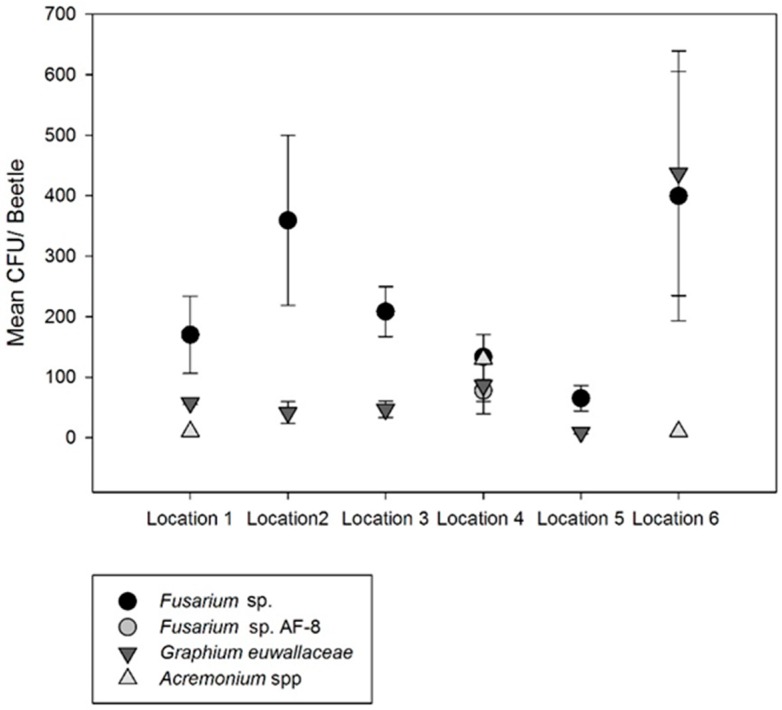
Estimated abundance (CFU) of fungal species recovered from 35 *E.* nr *fornicatus* at six locations within the south Florida avocado growing region.

Symbiotic fungi were found in all but four of the 35 beetles sampled and eleven fungal associates were identified including three yeast species. The unknown *Fusarium* sp. EF-1α sequence showed 99% identity match with *Fusarium ambrosium* GeneBank accession numbers KC691528. Similarly, sequences generated by the RBP2 primer set 7cf /11 showed 99% match with the *F. ambrosium* accession number KM406645. However, the sequence generated by the RBP2 primer set 5f2/7cr displayed 99% of identity with AF4 and no amplification was obtained for the RBP1 primer sets. The unknown *Fusarium* sp. was the most abundant and frequently found fungi in 28 of 35 beetles from all sampled sites. The number of *Fusarium* sp. colony forming units (CFU) per beetle ranged from 62–375 (Table 1, Figure 2). *Graphium euwallaceae—*identity based on 99% match of its 18s rRNA with the GeneBank accession number KF542895.1—was the second most frequent and abundant fungal associate isolated from 22 of the 35 sampled beetles from all locations, with a range of 20 to 82 CFUs/per beetle. *Acremonium* sp.—identity based on 99% match of its LSU sequence with the accession number KP030843.1—was recovered from three beetles in two locations with CFUs in the ranging from 10 to 130. One beetle from location 5 was carrying five CFUs of *Acremonium morum*, identified by its LSU with 99% identity with GeneBank accession FJ176880. In addition, one beetle was found carrying *Acrimonium masseei* that was identified by 99% identity of its LSU sequence with GeneBank accession number HQ232060. AF-8—of which EF-1α and RBP2 sequences showed 99% identity with GeneBank accession numbers KC691549.1 and KC691638.1—was isolated from two beetles carrying 60 to 95 CFUs each. AF-6 with EF-1α sequence showing 100% identity with the accession number KC691545 and RPB2 sequence with 99% of identity with the accession number KC691634 was only found in one branch sample at location 6 (Table 1). In addition, *Elaphocordyceps* sp.—identified based on its LSU 99% identity with gene bank accession number KM242367—was found associated with two *E.* nr. *fornicatus* beetles from two different locations carrying five CFUs each. The unknown *Fusarium* sp. was found as a single fungal symbiont in six out of 35 sampled beetles. *Graphium euwallaceae* was always found co-occurring with either *Fusarium* sp. (21 of 22 beetles) or with AF-8 (1 of 22 beetles). *Acremonium* species were always associated with beetles carrying *Fusarium* sp. and *G. euwallaceae.* AF-8 was found alone or together with *G. euwallaceae*. The yeast species identified based on their LSU sequences were: (1) *Zygozyma oligophage*—97% identity with GB accession number DQ518998—present in location 6 (3 of 10 beetles, 15 CFU/beetle), (2) *Hannaella* sp.—99% identity with GB accession number DQ518998KM206722—present at location 5 (1 of 5 beetles, 15 CFU/beetle), and (3) *Candida germanica*—98% identity with GB accession number DQ518998AF245401—present at location 4 (3 of 5 beetles, 28 CFU/beetle).

In addition to fungal associates, three bacteria species were found associated with adult *E.* nr. *fornicatus*. Bacterial isolates identified based on 16rRNA sequence include: (1) *Sphingomonas* sp.—99% identity with GB accession numbers DQ202286, HM241215 and KT984987—present at locations 4 (2 of 5 beetles, 45 CFU/beetle) and 6 (4 of 10 beetles 150 CFU/beetle); (2) *Ochrobactrum* sp.—99% identity with GB accession number KU902423—present at location 6 (2 of 10 beetles, 50 CFU/beetle), and (3) *Chryseobacterium* sp.—98% identity with GB accession numbers KR1896216 and KM229318—present at locations 5 (2 of 5, 20 CFU/beetle) and location 6 (1 of 10 beetles, 15 CFU/beetle).

## 4. Discussion

All beetles sampled from 33 locations within the avocado growing region matched *E*. nr. *fornicatus* sp. #2, which suggested a single introduction or multiple introductions of the same species into Florida. This species has now invaded the entire commercial avocado production area in Miami-Dade County. Little is known about the host range of *E*. nr. *fornicatus* sp. #2 in Florida. It was first reported in the state in 2002 attacking Royal Poinciana (*Delonix regia* (Boj. *ex* Hook.) Raf.; Fabales: Fabaceae), and 10 years later, was found attacking avocado trees [7]. In subsequent years, the beetle was detected in seven avocado orchards [10] and associated with dead branches of a mango tree at the University of Florida Tropical Research and Education Center at Homestead, Florida [24]. The widespread distribution of the beetle in the avocado growing region, and the finding of *E.* nr. *fornicatus* infestations in avocado at Fort Myers (Lee County, FL, USA) and in swampbay (*Persea palustris* (Raf.) Sarg.) trees at Lake Placid (Highlands County, FL, USA) approximately 250 km from the commercial production region, suggests that other host plants could be facilitating the spread of this beetle in Florida. In addition, this beetle can potentially spread through movement of infested nursery stock [25]. Species within the *E. fornicatus* complex have been reported to be highly polyphagous, attacking more than 300 host species in 58 plant families; including avocado in California and Israel [3,4,26,27]. There are three distinct races of avocados: Mexican, Guatemalan and West Indian. Some important commercial cultivars are hybrids of the various races. All previous reports of damage on avocado by *E.* nr. *fornicatus* species were on Guatemalan, Mexican, or Guatemalan-Mexican avocado hybrids, which are grown commercially in California and Israel. Here we report infestations on West Indian and West Indian-Guatemalan avocado hybrids grown in Florida. Affected varieties in the orchard with the detected outbreak include: “Donnie”, “Beta”, “Dupuis” “Monroe” and “Buck 3”. Donnie, the most abundant cultivar in the orchard, was more affected than the other cultivars in the grove. More studies are needed to determine avocado cultivar preferences by *E.* nr. *fornicatus* in Florida. The host plant range of *E.* nr. *fornicatus* in Florida warrants further attention.

Earlier work with *E.* nr. *fornicatus* was initiated when this beetle was first detected attacking avocado in Florida [7]. Kasson et al. [8] and O’Donnell et al. [2] reported that *E.* nr. *fornicatus* sp. #2 was associated with AF-8 and AF-6. These studies were conducted with a limited number of samples from the original detection site, and prior to populations reaching damaging levels. In this 2016 study, with a broader sample universe, an unknown *Fusarium* sp. was the most abundant and frequently found fungus associated with *E.* nr. *fornicatus*. Current identification of AF species is based in on EF-1α, ITS, RBP1 and RBP2 sequences. EF-1α is considered highly informative and instrumental to resolve all known species within the AFC [2]. *Fusarium* sp. isolates found in this study displayed 99% of identity with EF-1α and RBP2 primer set 7cf/11 of *Fusarium ambrosium*, but RPB2 primer set 5f2/7cr sequence showed similar percentage of identity with AF-4. Similarly, Kasson et al. [8] reported the existence of putative interspecific *F. ambrosium* hybrids with EF-1α, rDNA and RBP1 sequences identical to *F. ambrosium* but RBP2 sequence matching AF-4. EF-1α and partial RBP2 sequences support the dominant *Fusarium* sp. in this study as a member of AFC closely related to *F. ambrosium*. Further phylogenetic analysis and the identification of additional DNA sequence markers may be required for the recognition and diagnosis of this *Fusarium* sp. Overall the available evidence suggests that *E.* nr. *fornicatus* sp. #2 can be associated with at least three species of the AFC and that beetle–fusaria associations may not be obligate. Other studies have shown similar plasticity in ambrosia beetle–fungi associations [1]. In previous detections of *E.* nr. *fornicatus* associated with AF-8 and AF-6, the latter were not causing conspicuous damage to avocados. Here we document populations of this beetle associated with an unknown *Fusarium* sp. causing significant damage in two south Florida avocado orchards. This *Fusarium* sp. was prevalent in xylem lesions surrounding the beetle galleries unlike AF-8 and AF-6, which were seldom found associated with the lesions. Other fungi isolated from beetles (*Graphium euwallacea* and *Acremonium* spp.) were not recovered from lesions, and this suggests that they may grow confined mostly in beetle galleries and play a role in the beetle’s nutrition. The available evidence suggests that this unknown *Fusarium* sp. is the main fungus contributing to the damage of avocado in south Florida. The role of *Fusarium* sp. in the recent outbreak and its pathogenicity to avocados also warrants further research.

*Graphium euwallacea* was the second most frequent and abundant fungal associate of *E.* nr *fornicatus* in Florida. This fungus was reported in association with *E.* nr. *fornicatus* sp. #1 in Israel [27] and California [6]. Other *Graphium* sp. have been reported in association with *Euwallacea validus* (Eichhoff 1875) by O’Donnell et al. [2], who suggested an ancestral association between *Graphium* fungi and *Euwallacea* species. Our findings support this hypothesis but differ from the reports of pathogenicity of *G. euwallacea* to avocado [6]. In our survey, *G. euwallacea* was not recovered from any of the wood lesions surrounding the beetle galleries, and this suggests that the growth of this fungus is limited to the beetle galleries. According to Freeman et al. [27] *G. euwallacea* is the predominant symbiont in the initial stages of gallery formation and the main food source (i.e., dominant fungus) for larvae during their development. *Graphium euwallacea* is consumed during maturation of adult beetles when *F. euwallacea* becomes more readily available. Controlled experiments are needed to understand the dynamics and nutritional attributes of the fungal species throughout the life cycle of *E.* nr. *fornicatus* in Florida.

Three bacterial species were recovered from adult female *E.* nr. *fornicatus* in Florida. The bacteria most frequently observed were identified as *Sphigomonas* spp. (Sphigomonodales: Sphigomonodaceae), gram-negative bacteria in the α-4 subclass of the Protobacteria, known for their biodegradative capabilities, comprising cellulose aromatic compounds and lignin degradation [28]. They have been found associated with plant roots, and in some cases show antagonism against pathogenic fungi [29]. This characteristic could be associated with their ability to produce degradative enzymes such as chitinases as was reported for these bacterial species by Zhu et al. [30]. *Sphigomonas* also have been found as endophytes of two tree species—cottonwood (*Populus* sp.; Malpighiales: Saliaceae) and willow (*Salix* sp.; Malpighiales: Saliaceae)—showing nitrogen fixing capabilities in a symbiotic relationship that helps the plant survive under nutrient-poor conditions [31]. The second most common bacterial associate was identified as *Chryseobacterium* sp. (Flavobacteriales: Flavobacteriaceae), a gram-negative, non-motile, non-spore-forming, filamentous bacterium, and member of the heterogeneous group *Cytophaga-Flavobacterium-Bacteroides* [32]. *Chryseobacterium* sp. isolates from insect gut microbiota have been shown to produce wood degrading enzymes such as pectinase in longhorned beetles (Cerambycidae) [33], and cellulose degrading enzymes, cellobiohydrolase and β-glucosidase in termites [34]. The third bacterium identified belongs to the genus *Ochrobactrum* (Rhizobiales: Brucellaceae), in the α-2 subclass of the proteobacteria, a gram-negative non-spore forming, strictly aerobic bacterium [35]. *Octobractum* has been found in termite guts forming part of their microbiota that functions in hemicellulose degradation [36]. In general, studies have shown that insect-gut associated bacteria may play a role in insect nutrition, most likely by providing nitrogen and micronutrients, by detoxifying secondary plant compounds through enzymatic degradation and by “keeping clean” the ambrosia fungal culture through antagonistic effects against foreign fungal contaminants [37,38].

In addition, three yeast species were recovered from *E.* nr. *fornicatus* in Florida, including *Lipomyces oligophage* (Van der Walt & Arx) Kurtzman, Albertyn & Basehoar-Powers (Saccharomycetales: Lipomycetaceae), *Candida germanica* Kurtzman, Robnett & Yarrow (Saccharomycetales), and *Hannaella* sp. (Ascomycota: Schizosaccharomycotina: Tremelales). *Zygozyma oligophaga* (Saccharomycetales: Lipomycetaceae), the type species of the genus *Zygozyma,* has only been isolated from two frass samples: one from an unidentified bark beetle in a *Ficus trichpoda* Naker (Rosales: Moraceae), and one from the platypoid ambrosia beetle, *Crossotarsus externedentatus* Scheidl, infesting *Macaranga capensis* Benth (Malpighiales: Euphorbiaceae), both in South Africa [39,40]. The literature for this group only covers the basic description of the genera but provides no information regarding their functions as insect-associates. *Candida germanica* was isolated from the atmosphere over Germany [41] and no additional reports are available. Other *Candida* species have been isolated from the galleries, frass, and guts of different beetle species [42], and in many cases their extracellular enzymatic production profile links them with wood degradation [43]. The genus *Hannaella* comprises few species that are mainly phyllosphere-inhabiting yeasts [44]. *Hannaella* spp. have been isolated from the integuments of leafcutter ant males and fungus gardens of the leafcutter ants, where they are believed to function in plant material degradation, making nutrients available for the mutualistic fungus and the ants, as evidenced by the production of enzymes such as pectinases, polygalacturonases, lipases ligninases, and xylaneses. In addition, *Hannaella* spp. consumed galacturonic acid resulting from the degradation of the plant pectins, otherwise, toxic to the fungal symbiont [45]. Similar to the bacterial symbionts, yeast may have a role in nutrient availability and detoxification of deleterious compounds.

## 5. Conclusions

The recent outbreak of *E*. nr. *fornicatus* beetles in two avocado orchards in Florida causing extensive branch dieback warrants further attention by tropical fruit growers, entomologists, and plant pathologists. More research is needed to determine which factors trigger attacks by this beetle. The role of *Fusarium* sp. in the recent outbreak and its pathogenicity to avocados also warrants further research. While integrated pest management programs are developed for this pest beetle, avocado growers should periodically survey for trees showing branch dieback and signs of beetle attack at junctions of small and mid-size shaded branches showing the presence white “sugar volcanoes”. Infested branches should be removed and destroyed (chipped, burned or buried) to prevent further spread of these beetles. During our south Florida survey a large number of beetles were captured in locations with no apparent damage to the avocado trees suggesting that *E.* nr. *fornicatus* are associated with other host(s) outside the groves or with dead trees or branches inside the grove. Notably, most of the surveyed locations have had laurel wilt infections in the recent years, which could have influenced the abundance of *E.* nr. *fornicatus* by increasing the availability of dying and dead trees. More research is needed to determine the host range of *E.* nr. *fornicatus* and the potential threat that this beetle and its fungal associates pose to the avocado industry in south Florida.

## Figures and Tables

**Table 1:** Frequency of fungal isolates recovered from 35 beetle heads and the symptomatic tree tissue of 30 logs collected in six locations in the Florida avocado growing region.

Location Coordinates	Isolation Source	Isolate ID	Sample										Frequency n/N
			1	2	3	4	5	6	7	8	9	10	
Location 1 25°27′53″ N 80°31′6″ W	Plant tissue	*Fusarium* sp.	x	x	x	x	x						5/5
	Beetle	*Fusarium* sp.	x		x	x	x						4/5
		*G.* *euwallaceae*				x	x						2/5
Location 2 25°32′16″ N 80°29′11″ W	Plant tissue	*Fusarium* sp.	x	x	x	x	x						
	Beetle	*Fusarium* sp.	x	x	x	x	x						5/5
		*G.* *euwallaceae*	x	x	x	x							4/5
Location 3 25°32′28″ N 80°25′44″ W	Plant tissue	*Fusarium* sp.	x	x	x	x	x						
	Beetle	*Fusarium* sp.			x	x	x						3/5
		*G.* *euwallaceae*			x	x	x						3/5
		*A. masseei*					x						1/5
Location 4 25°29′8″ N 80°32′11″ W	Plant tissue	*Fusarium* sp.	x	x	x	x	x						
	Beetle	*Fusarium* sp.			x		x						2/5
		AF-8	x	x									2/5
		*G. euwallaceae*	x				x						2/5
		*Acremonium sp.*					x						1/5
		*Elaphocordyceps sp.*					x						1/5
Location 5 25°35′50″ N 80°30′16″ W	Plant tissue	*Fusarium* sp.	x	x	x	x	x						5/5
	Beetle	*Fusarium* sp.	x	x	x	x	x						5/5
		*G. euwallaceae*	x	x	x	x	x						5/5
		*A. morum*					x						1/5
Location 6 25°31′31″ N 80°30′16″ W	Plant tissue	*Fusarium* sp.	x	x	x								3/5
		AF-8				x							1/5
		AF-6					x						1/5
	Beetle	*Fusarium* sp.	x		x	x	x	x	x	x	x	x	9/10
		*G. euwallaceae*	x				x		x	x	x	x	5/10
		*Acremonium* sp.										x	1/10
		*Elaphocordyceps* sp.			x								1/10

## References

[1] Carrillo D., Duncan R.E., Ploetz J.N., Campbell A.F., Ploetz R.C., Peña J.E. (2014). Lateral transfer of a phytopathogenic symbiont among native and exotic ambrosia beetles. Plant Pathol..

[2] O’Donnell K., Sink S., Ran L.H.R., Hulcr J., Kasson M.T., Ploetz C.R., Konkol J.L., Ploetz J.N., Carrillo D., Campbell A. (2015). Discordant phylogenies suggest repeated host shifts in the *Fusarium–Euwallacea* ambrosia beetle mutualism. Fungal Genet. Biol..

[3] Eskalen A. (2013). Host range of Fusarium dieback and its ambrosia beetle (Coleoptera: Scolytinae) vector in Southern California. Plant Dis..

[4] Mendel Z., Protasov A., Sharon M., Zveibil A., Yehuda S.B., O’Donnell K., Rabaglia R., Wysoki M., Freeman S. (2012). An Asian ambrosia beetle *Euwallacea fornicatus* and its novel symbiotic fungus *Fusarium* sp. pose a serious threat to the Israeli avocado industry. Phytoparasitica.

[5] Freeman S., Sharon M., Maymon M., Mendel Z., Protasov A., Aoki T., Eskalen A., O’Donnell K. (2013). Fusarium euwallaceae sp. nov.—A symbiotic fungus of *Euwallacea* sp., an invasive ambrosia beetle in Israel and California. Mycologia.

[6] Lynch S.C., Twizeyimana M., Mayorquin J.S., Wang D.H., Na F., Kayim M., Kasson M.T., Thu P.Q., Bateman C., Rugman-Jones P. (2016). Identification, pathogenicity and abundance of *Paracremonium pembeum* sp. nov. and *Graphium euwallaceae* sp. nov. two newly discovered mycangial associates of the polyphagous shot hole borer (*Euwallacea* sp.) in California. Mycologia.

[7] Carrillo D., Duncan R., Peña J.E. (2012). Ambrosia beetles (Curculionidae: Scolytinae) that breed in avocado wood in Florida. Fla. Entomol..

[8] Kasson M.T., O’Donnell K., Rooney A.P., Sink S., Ploetz R.C., Ploetz J.N., Konkol J.L., Carrillo D., Freeman S., Mendel Z. (2013). Aninordinate fondness for Fusarium: Phylogenetic diversity of fusaria cultivated by ambrosia beetles in the genus *Euwallacea* on avocado and other plant hosts. Fungal Genet. Biol..

[9] Cooperband M.F., Stouthamer R., Carrillo D., Eskalen A., Thibault T., Cossé A.A., Castrillo L.A., Vandenberg J.D., Rugman-Jones P.F. (2016). Biology of two members of the *Euwallacea fornicatus* species complex (Coleoptera: Curculionidae: Scolytinae), recently invasive in the USA, reared on an ambrosia beetle artificial diet. Agric. For. Entomol..

[10] Carrillo D., Narvaez T., Cossé A.A., Stouthamer R., Cooperband M.F. (2015). Attraction of *Euwallacea* nr. *fornicatus* (Coleoptera: Curculionidae: Scolytinae) to lures containing quercivorol. Fla. Entomol..

[11] Carrillo D. (2015). Personal observation.

[12] De La Torre C. (2016). Personal Communication.

[13] United States Department of Agriculture, National Agricultural Statistics Service Census of Agriculture 2012. https://quickstats.nass.usda.gov/results/2126FF9A-4DBC-3F8E-9BB8-CA1976617919.

[14] United States Department of Agriculture, National Agricultural Statistics Service Census of Agriculture 2012. https://quickstats.nass.usda.gov/results/AA8D651A-9946-3384-8098-D0D9FDEAC0FC.

[15] Kendra P.E., Narvaez T.I., Montgomery W.S., Carrillo D. Ambrosia beetle communities in forest and agricultural ecosystems with laurel wilt disease. Proceedings of the 53rd Annual Meeting of the Entomological Society of America.

[16] Doyle J., Doyle J.L. (1987). Genomic plant DNA preparation from fresh tissue—CTAB method. Phytochem. Bull..

[17] Folmer O., Black M., Hoeh W., Lutz R., Vrijenhoek R. (1994). DNA primers for amplification of mitochondrial cytochrome C oxidase subunit I from diverse metazoan invertebrates. Mol. Mar. Biol. Biotechnol..

[18] Dole S.A., Jordal B.H., Cognato A.I. (2010). Polyphyly of *Xylosandrus* Reitter inferred from nuclear and mitochondrial genes (Coleoptera: Curculionidae: Scolytinae). Mol. Phylogenetics Evol..

[19] O’Donnell K., Kistler H.C., Cigelnik E., Ploetz R.C. (1998). Multiple evolutionary origins of the fungus causing Panama disease of banana: Concordant evidence from nuclear and mitochondrial gene genealogies. Proc. Natl. Acad. Sci. USA.

[20] Liu Y.J., Whelen S., Hall B.D. (1999). Phylogenetic relationships among ascomycetes: Evidence from an RNA polymerase II subunit. Mol. Biol. Evol..

[21] Vilgalys R., Hester M. (1990). Rapid genetic identification and mapping of enzymatically amplified ribosomal DNA from several Cryptococcus species. J. Bacteriol..

[22] White T.J., Bruns T., Lee S., Taylor J. (1990). Amplification and direct sequencing of fungal ribosomal RNA genes for phylogenies. PCR Protocols, a Guide to Methods and Applications.

[23] Marchesi J.R., Sato T., Weightman A.J., Martin T.A., Fry J.C., Hiom S.J., Wade W.G. (1998). Design and evaluation of useful bacterium-specific PCR primers that amplify genes coding for bacterial 16S rRNA. Appl. Environ. Microbiol..

[24] Carrillo D. (2016). Personal observation.

[25] Cooperband M.C. (2013). Personal communication.

[26] Danthanarayana W. (1968). The distribution and host-range of the shot-hole borer (*Xyleborus fornicatus* Eichh.) of tea. Tea Q..

[27] Freeman S., Sharon M., Dori-Bachash M., Maymon M., Belausov E., Maoz Y., Margalit O., Protasov A., Mendel Z. (2016). Symbiotic association of three fungal species throughout the life cycle of the ambrosia beetle *Euwallacea* nr. *fornicatus*. Symbiosis.

[28] Bugg T.D., Ahmad M., Hardiman E.M., Singh R. (2011). The emerging role for bacteria in lignin degradation and bio-product formation. Curr. Opin. Biotechnol..

[29] White D.C., Sutton S.D., Ringelberg D.B. (1996). The genus Sphingomonas: Physiology and ecology. Curr. Opin. Biotechnol..

[30] Zhu X., Zhou Y., Fenf J. (2007). Analysis of both chitinase and chitosanase produced by *Sphingomonas* sp. CJ-5. J. Zhejiang Univ. Sci..

[31] Doty S.L., Oakley B., Xin G., Kang J.W., Singleton G., Khan Z., Vajzovic A., Staley J.T. (2009). Diazotrophic endophytes of native black cottonwood and willow. Symbiosis.

[32] Vandamme P., Bernardet J.F., Segers P., Kersters K., Holmes B. (1994). New Perspectives in the Classification of the *Flavobacteria*: Description of *Chryseobacterium* gen. nov., *Bergeyella* gen. nov., and *Empedobacter* nom. rev.. Int. J. Syst. Evol. Microbiol..

[33] Park D., Oh H., Jeong W., Kim H., Park H., Bae K.S. (2007). A culture-based study of the bacterial communities within the guts of nine longicorn beetle species and their exo-enzyme producing properties for degrading xylan and pectin. J. Microbiol. Seoul.

[34] Cho M.J., Kim Y.H., Shin K., Kim Y.K., Kim Y.S., Kim T.J. (2010). Symbiotic adaptation of bacteria in the gut of *Reticulitermes speratus*: Low endo-β-1, 4-glucanase activity. Biochem. Biophys. Res. Commun..

[35] Swings J., Lambert B., Kersters K., Holmes B., Falkow S., Rosenberg E., Schleifer K.H., Stackebrandt E. (2006). The genera *Phyllobacterium* and *Ochrobactrum*. The Prokaryotes, a handbook on the biology of bacteria, ecophysiology, isolation, identification and applications.

[36] Schäfer A., Konra R., Kuhnigk T., Kämpfer P., Hertel H., König H. (1996). Hemicellulose-degrading bacteria and yeasts from the termite gut. J. Appl. Bacteriol..

[37] Calderón-Cortés N., Quesada M., Watanabe H., Cano-Camacho H., Oyama K. (2012). Endogenous plant cell wall digestion: A key mechanism in insect evolution. Annu. Rev. Ecol. Evol. Syst..

[38] Oliver K.M., Degnen P.H., Burke G.R., Moran N.A. (2010). Facultative symbionts in aphids and the horizontal transfer of ecologically important traits. Annu. Rev. Entomol..

[39] Van der Walt J.P., Von Arx J.A., Ferreira N.P., Richards P.D.G. (1987). *Zygozyma*, a new genus of the Lipornycetaceae. Syst. Appl. Microbiol..

[40] Van der Walt J.P., Wingfield M.J., Yamada Y. (1990). *Zygozyma smithiae* sp. n. (Lipomycetaceae), a new ambrosia yeast from Southern Africa. Antonie Van Leeuwenhoek.

[41] Kurtzman C.P., Robnett C.J., Yarrow D. (2001). Two new anamorphic yeasts: *Candida germanica* and *Candida neerlandica*. Antonie Van Leeuwenhoek.

[42] Suh S.O., Nguyen N.H., Blackwell M. (2008). Yeasts isolated from plant-associated beetles and other insects: Seven novel *Candida* species near *Candida albicans*. FEMS Yeast Res..

[43] Yun Y.H., Suh D.Y., Yoo H.D., Oh M.H., Kim S.H. (2015). Yeast Associated with the Ambrosia Beetle, *Platypus koryoensis*, the Pest of Oak Trees in Korea. Mycobiology.

[44] Landell M.F., Brandão L.R., Barbosa A.C., Ramos J.P., Safar S.V., Gomes F.C., Sousa F.M., Morais P.B., Broetto L., Leoncini O. (2014). *Hannaella pagnoccae* sp. nov., a tremellaceous yeast species isolated from plants and soil. Int. J. Syst. Evol. Microbiol..

[45] Arcuri S.L., Pagnocca F.C., da Paixão Melo W.G., Nagamoto N.S., Komura D.L., Rodrigues A. (2014). Yeasts found on an ephemeral reproductive caste of the leaf-cutting ant *Atta sexdens rubropilosa*. Antonie Van Leeuwenhoek.

